# Ultrasound radiomics and genomics improve the diagnosis of cytologically indeterminate thyroid nodules

**DOI:** 10.3389/fendo.2025.1529948

**Published:** 2025-02-28

**Authors:** Lu Chen, Mingbo Zhang, Yukun Luo

**Affiliations:** Department of Ultrasound, The First Medical Center of Chinese People’s Liberation Army (PLA) of China General Hospital, Beijing, China

**Keywords:** ultrasonography, radiomics, genomics, molecular testing, indeterminate thyroid nodule, high throughput sequencing, assisted diagnosis

## Abstract

**Background:**

Increasing numbers of cytologically indeterminate thyroid nodules (ITNs) present challenges for preoperative diagnosis, often leading to unnecessary diagnostic surgical procedures for nodules that prove benign. Research in ultrasound radiomics and genomic testing leverages high-throughput data and image or sequence algorithms to establish assisted models or testing panels for ITN diagnosis. Many radiomics models now demonstrate diagnostic accuracy above 80% and sensitivity over 90%, surpassing the performance of less experienced radiologists and, in some cases, matching the accuracy of experienced radiologists. Molecular testing panels have helped clinicians achieve accurate diagnoses of ITNs, preventing unnecessary diagnostic surgical procedures in 42%–61% of patients with benign nodules.

**Objective:**

In this review, we examined studies on ultrasound radiomics and genomic molecular testing for cytological ITNs conducted over the past 5 years, aiming to provide insights for researchers focused on improving ITN diagnosis.

**Conclusion:**

Radiomics models and molecular testing have enhanced diagnostic accuracy before surgery and reduced unnecessary diagnostic surgical procedures for ITN patients.

## 1 Introduction

The global incidence of thyroid cancer has risen significantly over the past 30 years, now comprising 3.4% of all annual cancer diagnoses worldwide (1), making it the eighth most estimated new cancer worldwide (2). According to the American College of Radiology Thyroid Imaging Reporting and Data System (ACR TI-RADS) (3), ultrasound-guided fine-needle aspiration (FNA) cytology is required for most suspicious thyroid nodules. However, 20%–30% of FNA samples yield indeterminate results (4), termed cytologically indeterminate thyroid nodules (ITNs). These include Bethesda categories III, IV, and V nodules, which are graded using the Bethesda System for Reporting Thyroid Cytopathology (5). If a thyroid nodule is categorized as atypia of undetermined significance (Bethesda III), follicular neoplasm (Bethesda IV), or suspicious for malignancy (Bethesda V), the risk of malignancy is 13%–30%, 23%–34%, and 67%–83%, respectively (5). More than half of the patients with ITNs undergo diagnostic surgery (6), with a high morbidity of thyroidectomy in general and the increasing medical costs for unnecessary surgical resection of benign ITNs. Among the excised nodules, 60%–80% of them are benign upon final pathological analysis (7–9). Ultrasonography (US) is the first-line imaging tool for detecting thyroid nodules. Radiologists assess nodules using ACR TI-RADS features (3), including composition, echogenicity, shape, margin, and echogenic foci. However, grayscale ultrasound assessment for ACR TI-RADS 4 or 5 nodules (TR4 and TR5) has low diagnostic specificity (44%–67.3%) (10–12) and high interobserver variability (11, 13, 14), which contribute to the high rate of indeterminate FNA results. Therefore, the current clinical challenge lies in improving preoperative diagnostic accuracy to avoid unnecessary surgical procedures for benign ITNs, potentially through enhanced follow-up or minimally invasive approaches.

Omics technologies offer new insights into the preoperative diagnosis of ITNs. Radiomics refers to the process of transforming multiple medical imaging data into a large amount of quantitative data beyond visual interpretation using artificial intelligence (AI) to predict clinical diagnosis, disease risk, and prognosis (15). Sources of data for radiomics include conventional B-mode ultrasound (BMUS) images (16, 17), contrast-enhanced ultrasound (CEUS) videos (18, 19) and shear-wave elastography (SWE) images (20). Many AI-assisted diagnostic models show high accuracy (16, 17, 21), lower intraobserver variability (16, 22), and a reduced rate of unnecessary FNA procedures (23, 24). To some extent, molecular testing assists in the preoperative diagnosis of ITNs. The somatic BRAF V600E (c.1799T>A) mutation shows 100% specificity for thyroid carcinoma, which eliminates some secondary surgery for ITNs (25, 26). However, mutant BRAF V600E occurs in 50%–80% of cancers (27). The retrovirus-associated DNA sequence (RAS) gene is the most common genetic alteration in ITNs but is less specific to TNs with follicle-patterned lesions (28). Genomics has taken advantage of hundreds of genetic alterations to assist in the diagnosis of ITNs by testing for point mutations, gene expression, gene fusion, and copy number alterations (29–31). These panels serve as supplementary tools for ITN diagnosis and can reduce overtreatment rates.

In many studies, creative radiomic models and molecular testing panels have enhanced the diagnostic accuracy of ITNs. However, many AI-assisted radiomics models and molecular testing panels remain in the preliminary or validation phase and require further external validation and optimization. Therefore, we reviewed recent publications on ITN diagnosis (mostly TR4/TR5 nodules) from a radiomic or genomic perspective. Future research should focus on making these approaches more cost-effective and scalable, ultimately benefiting patients and clinicians in routine clinical practice.

## 2 Radiomics for ITN diagnosis

### 2.1 Diagnostic models based on BMUS features

In clinical practice, US is recommended as the primary imaging tool for assessing TNs (7). According to the well-established ACR TI-RADS (3), radiologists recommend five US features (composition, echogenicity, shape, margin, and echogenic foci) as diagnostic criteria to assess the risk grades of TNs. Although microcalcifications are an independent risk factor for malignancy in ITNs (32), hypoechoic features (p=0.014) and calcifications (p=0.019) are strong predictors of thyroid cancer with a two-fold increased risk of malignancy for ITNs (33). However, the evidence is inadequate for accurately evaluating ITNs in clinical practice, as ACR TI-RADS (3) and other US-based systems (7, 34, 35) depend on limited morphological features. Radiomics models that rely on BMUS features have shown superior diagnostic efficacy than models based solely on traditional US risk stratification systems (23, 24). Multimodality radiomics models are an alternative diagnostic choice for differentiating benign ITNs from malignancies preoperatively. The following sections focus on the potential of these models in clinical applications, along with details on the algorithms, software, and architectures used (**Table 1**).

**Table 1:** The main ultrasound radiomics studies on thyroid nodules diagnosis in Technique order.

Reference	Published year	Technique	No. of Subjects	Features	Method	Classifier	Main Performance
Park et al. (23)	2020	US	1624 TNs	first-order statistics, Texture (GLCM, GLRLM, histogram), wavelet	Rad_Score TIRADS	in-house texture analysis algorithms, LASSO	AUC:0.85 in training set AUC:0.75 in test set At Rad_5%: Sensitivity 95.6%, NPV 96.6% Specificity 33.1%, PPV 27.5%
Huang et al. (36)	2021	US	451 TNs	first-order statistics, GLCM, GLDM, GLRLM, GLSZM, NGTDM	ACR-Rad nomogram	LASSO, Linear Dependence analyses	AUC:0.877 in training set AUC: 0.864 in validation set
Wu et al. (16)	2021	US	2082 TR4-5 TNs	—	ResNet-50 model	ResNet-50, InceptionResnet v2, Desnet-121	AUC: 0.829 Sensitivity 0.790 Specificity 0.779
Peng et al. (37)	2021	US	22354 images	—	ThyNet model	a combined architecture of ResNet, ResNeXt, and DenseNet	AUC: 0.922
Matti et al. (38)	2021	US	88 ITNs	first‐order statistics features, textural (GLCM), statistical feature matrix	ResNe-50 model	ThyNet ResNet‐50 RF	ResNet‐50: AUC: 0.740
Han et al. (39)	2021	US	454 TNs	composition, echogenicity, orientation, margins, spongiform status, shape, calcifications	S-Detect 2 CAD system	S-Detect 2 CAD system	Sensitivity 81.4% Specificity 81.9% Accuracy 81.7%
Luo et al. (40)	2021	US	394 TNs	Vertl-RLNonUni, Vertl-GLevNonU, WavEnLH-s4 and WavEnHL-s5	Rad_score+TIRADS	LASSO	AUC: 0.889
Liang et al. (17)	2021	US	138 TNs	Echogenic foci, Margins, Composition, Echogenicity	AI-Sonic computer-aided design (CAD) system	deep learning	AUC: 0.919
Tang et al. (41)	2022	US	328 TNs	—	US Radiomics nomogram	LASSO	AUC: 0.651
Chen et al. (42)	2022	US	194 ITNs	composition, echogenicity, margins,shape, echogenic foci, nodule size, age, sex	SVM	SVM	Sensitivity 93.8% Specificity 56.5% PPV 60.0% NPV 92.9% Accuracy 71.8%
Kim et al. (43)	2022	US	16009 images	—	Three deep learning models	VGG16 VGG19 ResNet	Internal: VGG16_AUC 0.86 ResNet50_AUC 0.85 VGG19_AUC 0.83 External: VGG16_AUC 0.83 ResNet50_AUC 0.80 VGG19_AUC 0.81
Xavier et al. (44)	2022	US	105 images	size, shape, texture	BANN model	BANN	AUC: 0.88
Wang et al. (45)	2022	US	1007 TNs	—	ITS 100 system	Dynamic AI based on CNN	Sensitivity 92.21% Specificity 83.20% Accuracy 89.97%
Grégoire et al. (21)	2023	US	1335 ITNs	LR: age, hyperthyroidism, TN hardness, the French TI-RADS score, disrupted rim macrocalcifications, exclusively solid, Bethesda category RF: Bethesda category, French TI-RADS score, cytological subclassification, the number of typical cytonuclear abnormalities of PTC, TN hardness	LR model RF model	LR, RF	LR model AUC: 0.840 RF model AUC: 0.826
Zhou et al. (46)	2023	US	159 ITNs	composition, echogenicity, orientation, margin, shape and spongiform status	S-Detect unit	S-Detect software	AUC: 0.795
Wu et al. (47)	2024	US	1161 TNs	composition, echogenicity, shape, margin, Echogenic fogi	image-to-text -ChatGPT 4.0	ChatGPT 4.0+image-to-text CNN model	AUC: 0.83
Guo et al. (48)	2021	BMUS+CEUS	123 TNs	first-order statistics, textures, GLCM, grayscale tour matrix, grayscale region size matrix, domain grayscale difference matrix, and morphological features	BMUS + CEUS radiomics model	logistic regression analysis, LASSO	AUC: 0.861
Gong et al. (18)	2023	CEUS	148 TR4 TNs	location, shape, size, internal echo, border edge, calcification	AI-SONIC™ Thyroid and CEUS	AI-SONIC™ Thyroid (DEMETICS)	Sensitivity 96.61% Specificity 75.28% Accuracy 83.78% PPV 72.15% NPV 97.10%
Ren et al. (19)	2024	BMUS+CEUS	312 TR4-5 TNs	Morphology, intensity, textures, wavelet	BMUS+CEUS Radiomics nomogram	LASSO, multivariate logistic regression analysis	AUC: 0.851
Zhao et al. (24)	2020	US+SWE	849 TNs	contour, shape, textural phenotype, histogram, GLCM, GLRLM, GLSZM, NGTDM, GLDM, LBP, ect.	ML-assisted US+SWE visual Approach, US+SWE radiomics approach	DT, NB, KNN, LR, SVM, KNN-based bagging, RF, xgboost, multi-layer perception, gradient boosting tree	ML‐assisted US+SWE visual approach: AUC:0.953 US+SWE radiomics approach: AUC:0.882

AUC, area under the curve; B-US/BMUS, B-mode ultrasound; BANN, Bayesian artificial neural networks; CEUS, contrast-enhanced ultrasound; CNN, convolutional neural network; DT, Decision Tree; GLCM, gray-level co-occurrence matrix; GLDM, gray-level dependence matrix; GLRLM, gray-level run-length matrix; GLRM, gray-level co-occurrence matrix; GLSZM, gray-level size zone matrix; ITS100 system, Ian Thyroid Solution 100; KNN, k-Nearest Neighbor; LASSO, the least absolute shrinkage and selection operator logistic regression algorithm; LBP, Local Binary Pattern; LR, logistic regression; NB, Naive Bayes; NGTDM, neighboring gray tone difference matrix; NPV, negative predictive values; PPV, positive predictive values; RF, Random Forest; SVM, Support Vector Machines; SWE, shear-wave elastography; US, ultrasound.

#### 2.1.1 Assisted diagnostic models using machine learning

Machine learning (ML) is an AI method used to develop diagnostic models by training them on a set of pathologically confirmed nodules with labeled regions of interest. ML-based models for TN diagnosis have achieved AUC values ranging from 0.651 to 0.889 (23, 36, 40–42), supporting their use in recognizing benign TNs and reducing unnecessary surgical procedures. Chen et al. developed a support vector machine model to identify benign ITNs in 180 patients with confirmed pathology, achieving a sensitivity of 93.8% and a specificity of 56.5%. The negative predictive values (NPV) of the models for Bethesda III nodules were 93.9% and 93.8%, respectively. The authors believe that the high NPV value could support the ultrasound-guided follow-up of AI-based benign ITNs during surgery (42). Similarly, Keutgenon et al. developed a model using imaging data from 162 ITNs, achieving an AUC of 0.67 for differential diagnosis (44). In an independent test set of 19 ITNs, the method distinguished ITNs with an AUC value of 0.88 (p<0.001) (44).

When combined with well-known clinical, biological, and cytological information in a nomogram format, the multivariate logistic regression model achieved 90% specificity, 57.3% sensitivity, 73.4% PPV, 81.4% NPV, and an AUC of 0.840 (21). A random forest model achieved comparable results, with 87.6% specificity, 54.7% sensitivity, 68.1% PPV, 80% NPV, and an AUC of 0.826 (21). Both ML models enabled radiologists to stratify ITNs into low-, intermediate-, and high-risk categories (<6%, 6%–30%, and >30%, respectively) for malignancy (21).

#### 2.1.2 Assisted diagnostic models using deep learning

Deep learning (DL) models, which require larger datasets of primary US images than ML models, self-learn to recognize, locate, and predict the nature of TNs. These models require more original medical images than ML models and exhibit a higher AUC (0.740–0.970) (16, 17, 37, 38, 43, 46, 49), assisting clinicians in identifying benign TNs and reducing unnecessary surgical procedures. The diagnostic performance of radiomics models varies depending on the classifier used. Three DL models based on Visual Geometry Group 16 (VGG 16), VGG19, and ResNet methods exhibited superior diagnostic performance with an AUC of 0.83–0.86 than radiologists with an AUC of 0.71–0.76 (p<0.05). The VGG16 model demonstrated the highest diagnostic performance in both the internal (AUC 0.86; sensitivity, 91.8%; specificity, 73.2%) and external (AUC 0.83; sensitivity, 78.6%; specificity, 76.8%) test sets, although no significant differences were observed in AUCs among the three DL models (43). Wu et al. compared DL models basing on ResNet-50, Inception-ResNet V2, and Desnet-121 and found that the ResNet-50 model achieved the highest AUC values of 0.904, 0.845, and 0.829 for TR4, TR5, and TR4–5 nodules, respectively. The ResNet-50 model enhanced the diagnostic rate of malignancy from benign TR4 and TR5 TNs despite no significance in sensitivity and specificity compared with those of radiologist values in the current dataset (16). The ThyNet model, combining the architectures of the ResNet, ResNeXt, and DenseNet classifiers, was trained on over 10,000 images from 8,339 patients and reached an AUC of 0.922 for less challenging and unequivocal TNs (37). Training data affect AI model diagnostic performance. For example, using 88 Bethesda III nodules with final pathology, ThyNet showed an overall accuracy of 0.64 (38). ImageNet, which was trained on challenging ITN images, demonstrated an accuracy of 0.74 (38). Dynamic AI computer-assisted diagnostic systems have been developed from tens of thousands of determinate TN images, showing significantly higher specificity, PPV, and accuracy than the preoperative ultrasound ACR TI-RADS or C-TIRADS, such as the ITS100 system (p<0.001) (45) and S-Detect system (p<0.05) (46). Moreover, the ITN100 system achieved a sensitivity of 96.58% and an accuracy of 94.06%, comparable with FNA values (45).

In practice, AI models should be viewed as assistive tools for reducing workload and improving diagnostic accuracy rather than as standalone decision-makers, such as radiologists, endocrinologists, or surgeons. To assist clinicians in the management of thyroid nodules, a ThyNet-assisted strategy was proposed and tested in real-world clinical settings. The strategy improved the AUC of radiologists from 0.837 to 0.875 (p<0.0001) and from 0.862 to 0.873 (p<0.0001) in clinical tests (37). In the simulated setting, the rate of false negatives decreased from 61.9% to 35.2% using the ThyNet-assisted strategy, while the rate of missed malignancies decreased from 18.9% to 17.0% (37). Clinicians using and supervising AI-assisted models can enhance diagnostic accuracy in medical practice. However, AI models are not always correct, and it is essential for clinicians that they need to scrutinize their findings. For junior radiologists, in particular, it is critical to carefully consider various US features identified by AI in making the final diagnosis. These features include solid or mostly solid nodules; hyperechoic or isoechoic, hypoechoic, or very hypoechoic nodules; nodules with diverse shapes and margins; absence or presence of large comet-tail artifacts; macrocalcifications; punctate echogenic foci; nodules measuring ≥ 5 mm; and all parenchymal backgrounds (50). Each of these features has been associated with improved sensitivity (all p<0.004) and specificity (all p<0.001) (50).

### 2.2 Dual-modality radiomics-assisted diagnosis

Researchers have explored the use of dual-modality radiomics models, incorporating CEUS and SWE, to enhance the accuracy of differentiating benign from malignant thyroid nodules beyond BMUS data alone. CEUS serves as a complementary modality to BMUS by assessing the blood flow of TNs and demonstrating excellent sensitivity and specificity in discriminating between TNs. The AI-SONIC™ Thyroid intelligent diagnosis system is based on BMUS images with an accuracy of 83.02% (18). When combined with CEUS, the AI-SONIC™ Thyroid system showed significantly higher sensitivity, NPV, and AUC (0.859) than the AI-SONIC™ Thyroid system or CEUS alone (p<0.05), indicating that the combination of US and CEUS is beneficial for the early detection of malignant TNs (18). Guo et al. found that their BMUS- and CEUS-based models (AUC, 0.861) were significantly superior to the BMUS-only model (AUC, 0.791) and CEUS-only model (AUC, 0.766) (both p<0.05) (48). Another BMUS and CEUS dual-modal radiomics nomogram involving six variables (BMUS Rad-score, CEUS Rad-score, age, shape, margin, and enhancement direction) exhibited excellent calibration and discrimination in the training (n=219) and validation (n=93) cohorts, with AUCs of 0.873 and 0.851, respectively. This approach reduced the need for FNA from 35.3% to 14.5% and from 41.5% to 17.7% for TR4–5 TNs compared with ACR TI-RADS (19). Although CEUS can reveal vascular dynamic perfusion and enhancement patterns, its reliance on additional contrast agents (51) may be costly, and overlapping features in benign and malignant TNs can sometimes limit clinical applications (52). Superb microvascular imaging is an economical and noninvasive vascular imaging method with no contraindications and is comparable with CEUS in evaluating peripheral blood flow for malignant TN diagnosis (53).

SWE is another noninvasive technique that can be used to assess the mechanical properties of tissue elasticity to evaluate TNs. For patients with ITNs, a muscle deformation ratio greater than 1.53 kPa indicated a higher malignancy risk (AUC, 0.98) (54). Some studies suggest that combining BMUS and SWE could enhance diagnostic specificity for predicting thyroid malignancies (55). Zhao et al. (24) extracted six US features (size, composition, echogenicity, shape, margin, and echogenic foci) and five SWE parameters (SWE-mean, SWE-min, SWE-max, SWE-SD, and SWE-ratio) to build an AI-assisted visual model. This model demonstrated superior diagnostic performance than US alone, with an AUC of 0.951 vs. 0.900 for the validation dataset and 0.953 vs. 0.917 for the test dataset. When applying the US-added SWE visual radiomics model, the unnecessary FNA rate decreased from 30.0% to 4.5% in the validation dataset and from 37.7% to 4.7% in the test dataset, compared with ACR TI-RADS values (24).

## 3 Genomic molecular testing for ITN diagnosis

Molecular testing is an promising adjunctive tool in cancer diagnostics, offering advantages such as enhanced diagnostic accuracy and faster screening for TNs (56). This testing can provide additional diagnostic information for ITNs, even in the absence of indicative BMUS features (57), leading to its widespread adoption in clinical settings across some countries and regions to aid in the diagnosis of TNs. Available molecular testing panels for ITN diagnosis have evolved from single genes (e.g., B-type RAF kinase [BRAF] V600E) (27, 41, 58) to multiple genes (e.g., seven-gene group) (59–61) or genomic markers (29, 30, 62) [e.g., Afirma Gene Expression Classifier (GEC) (63)]. This section reviews the research on genetic markers for the diagnosis of ITNs. The details of the markers, platform, and performance of each molecular testing method are listed in **Table 2**.

**Table 2:** The main studies on ITNs diagnosis by multiple molecular testing.

Reference	Published Year	No. of Subjects	Method	Biomarkers	Main Performance
Patel et al. (9)	2018	191 Bethesda III or IV nodules	GSC	10,196 genes, 7 other components: a parathyroid cassette, a medullary thyroid cancer (MTC) cassette, a BRAFV600Ecassette, RET/PTC1 and RET/PTC3 fusion detection modules, follicular content index, Hürthle cell index, and Hürthle neoplasm index	Sensitivity 91% Specificity 68% NPV 96% PPV 47%
Endo et al. (64)	2020	289 Bethesda III or IV nodules	GEC or GSC	—	Younger age, larger nodule size, presence of Afirma suspicious nodule other than the index nodule and compressive symptoms were associated with a higher rate of surgery.
Hu et al. (65)	2021	50644 Bethesda III-VI nodules	GSC and XA	905 genomic variants and 235 fusion pairs from 593 genes (XA)	PPV of genes in Bethesda III/IV: ALK 60% BRAF 76% NTRK 96% RET fusions 100%
White et al. (66)	2022	280 Bethesda III or IV nodules	GEC or GSC	—	1 of 14 negative nodules demonstrating minimally invasive follicular carcinoma; 81 of 97 negative nodules were safe to undergo follow-up.
Ahmadi et al. (67)	2024	834 Bethesda III or IV nodules	GSC	10,196 genes, 7 other components	For Bethesda III/IV: Sensitivity 95%/94% Specificity 30%/42% NPV 89%/87% PPV 50%/65%
Steward et al. (29)	2018	286 Bethesda III or IV nodules	ThyroSeq v3	112 gene include a broad range of thyroid cancer-related point mutations, gene fusions, copy number alterations and gene expression alterations	Sensitivity 94% Specificity 82% NPV 97% PPV 66%
Nikiforova et al. (8)	2018	238 tissue samples and 175 FNA samples Of Bethesda III and V nodules	ThyroSeq v3	112 genes	Sensitivity 98.0% Specificity 81.8% Accuracy 90.9%
Carty et al. (68)	2020	405 Bethesda IV nodules	ThyroSeq v2 or 3	—	MT use for Bethesda IV nodules increased the surgical yield of cancer by 4-fold, identified all potentially aggressive malignancies, and allowed safe nonoperative surveillance for >80% of MT-negative patients.
Desai et al. (69)	2020	415 Bethesda III and V nodules	ThyroSeq v3	112 genes include a broad range of thyroid cancer-related point mutations, gene fusions, copy number alterations and gene expression alterations	Sensitivity 92.9% Specificity 90.3% NPV 98.3% PPV 67.7%
Livhits et al. (70)	2020	372 Bethesda III or IV nodules	ThyroSeq v3;GSC	RNA+DNA; RNA	Performance of GSC/ThyroSeq: Sensitivity 100%/97% Specificity 80%/85% NPV 54%/63% PPV 53%/63%
Torrecillas et al. (31)	2021	89 Bethesda III or IV nodules	Thyroseq v2/v3	—	Sensitivity 100% Specificity 65% NPV 100% PPV 37%
Yang et al. (71)	2024	134 Bethesda III-V nodules	ThyroSeq v3	112 genes	Sensitivity 89.6% Specificity 73.7% NPV 84.0% PPV 82.1%
Daniels et al. (60)	2020	118 Bethesda III or IV nodules	custom panel	23 genes (AKT1,APC, AXIN1, BRAF, CDKN2A, CTNNB1, DNMT3A, EGFR, EIF1AX, GNAS, HRAS, IDH1, KRAS, NDUFA13, NRAS, PIK3CA, PTEN, RET, SMAD4, TERT, TP53, TSHR, VHL)	The ACR TI-RADS classification system performs with low inter-reader and intra-reader reliability when assessing the genetic risk of ITNs.
Tolaba et al. (61)	2022	112 Bethesda III- IV nodules	7-gene mutation panel	BRAF and RAS (H/N/K) and the gene fusions PAX8/PPARG, RET/PTC1 and RET/PTC2	Sensitivity 86% Specificity 87% NPV 94% PPV 54%
Xu et al. (72)	2022	550 Bethesda III and VI nodules	Quantitative chromogenic imprinted gene *in situ* hybridization (QCIGISH)	SNRPN and HM13	for Bethesda III-IV: Sensitibvity 100% Specificity 92.3% PPV 96.6% NPV 100% Accuracy 97.5%
Hu et al. (62)	2022	140 ITNs	Thyroeva™	DNA (140 amplicons), RNA fusion (36 amplicons) and mRNA (169 amplicons)	Sensitivity 96% Specificity 93% Accuracy 95% AUC 0.94
Stewardson et al. (30)	2023	615 Bethesda III or IV nodules	ThyroSPEC (MassARRAY)	139 most prevalent mutations and gene fusions	Sensitivity 72% Specificity 70-78% NPV 76-91% PPV 46-65%
Zhou et al. (59)	2023	529 Bethesda III-V nodules	Customized NGS panel	14 thyroid cancer-related genes (AKT1, BRAF, CTNNB1, EIF1AX, HRAS, KRAS, NRAS, PAX8, PIK3CA, PTEN, RET, TERT, THADA, and TP53) and 21 types of gene rearrangements occurring in thyroid cancer (ACBD5, AFAP1L2, ALK, ATG10, BRAF, CALM2, CCDC6, ERC1, ETV6, FLNC, FMNL2, KIAA1217, KIAA1594, KIF20B, NCOA4, NTRK3, PAK1, PAX8, PIBF1, PPAR γ, PXK, RALGAPA2, RET, SND1, and STRN).	Sensitivity 97.8% Specificity 82.7% NPV 67.7% PPV 99.0%

GEC, Afirma genomic expression classifier; GSC, Afirma genomic sequencing classifier; ITN, indeterminate thyroid nodules; NPV, negative predictive value; PPV, positive predictive value; XA, Afirma Xpress.

### 3.1 Single-gene molecular testing

The BRAF V600E gene mutation, strongly associated with papillary thyroid carcinoma, is widely used as a biomarker for TN diagnosis in clinical practice (3). BRAF V600E mutations are more prevalent in Bethesda III nodules with cytological or architectural atypia (73), making this gene superior to RAS mutations in the diagnosis of thyroid cancer (59). Although RAS mutations are the most common genetic alteration in ITNs (74), many resected RAS-mutant nodules are benign, and most ITNs with RAS mutations tend to remain stable over time. Therefore, it is important to consider all RAS-mutant ITNs when avoiding immediate surgical resection (75). KRAS-mutant Bethesda IV nodules have a 50% risk of malignancy, and diagnostic surgery is recommended (76). PTEN and PAX8-PPARγ2 are regarded as low-risk alterations and are more prevalent in ITNs with architectural atypia (73). Patients with STRN-ALK fusion-positive nodules should undergo thyroid lobectomy because these nodules are usually malignant (77). Approximately 77% of THADA-IGF2BP3 fusion-positive thyroid nodules are malignant and require surgery (78). However, single-gene testing is sometimes inadequate for ITN diagnosis because the prevalence of BRAF V600E is low (2% in the ITN cohort) (65). Expanded molecular testing identified at least one more mutation in 44% of ITNs that were Afirma gene sequencing classifier (GSC) suspicious subjects (65).

### 3.2 Multiple-gene molecular testing

A seven-gene testing study by Tolaba et al. reported good performance, with sensitivity, specificity, PPV, and NPV of 86%, 77%, 54%, and 94%, respectively, in 112 FNA samples from patients with Bethesda III–V nodules, indicating a potential reduction in surgical procedures by 48% (61). Multiple-gene testing can also be used to assess genetic risk stratification for ITNs (59). Among the 529 Bethesda III–V nodules, 2 cases (0.44%) were categorized into the high-risk group, 426 cases (94.67%) were categorized into the BRAF-like group with histopathologic papillary patterned tumors, and 22 cases (4.89%) were categorized into the RAS-like group. These studies highlighted that multiple genes can be incorporated into the clinical diagnostic process of ITN management (59). Notably, the current ACR TI-RADS classification system has low inter- and intra-reader reliability when assessing the genetic risk of ITNs (60).

Two epigenetically imprinted genes, small nuclear ribonucleoprotein polypeptide N (SNRPN) and minor histocompatibility antigen H13 (HM13), were visualized and quantified using a quantitative chromogenic imprinted gene *in situ* hybridization (QCIGISH) method (79). The research team found an excellent performance of SNRPN and HM13 for Bethesda III–V nodules with a PPV of 97.8% and NPV of 100%, achieving a diagnostic accuracy of 98.2% as well as a PPV of 96.6% and an NPV of 100%, with a diagnostic accuracy of 97.5% for Bethesda III–IV nodules (72). This novel method based on imprinted biomarkers provides new insights into the effective distinction between malignant and benign TNs. The high PPV and NPV make QCIGISH an excellent diagnostic tool for both rule-in and rule-out thyroid nodules (72). These multiple-gene tests could improve the diagnosis of ITNs and reduce the need for diagnostic surgery. However, they are not suitable for Hürthle cell adenomas or carcinomas or noninvasive follicular thyroid neoplasms with papillary-like nuclear features.

### 3.3 Genomic molecular testing panels

The Afirma GSC (9) is an RNA-Seq testing panel designed for model prediction with over ten thousand genes and rare subgroups of the TN category, including parathyroid, medullary thyroid cancer, follicular content, Hürthle cells, and Hürthle neoplasms (9). In total, GSC enabled the accurate differentiation of benign Bethesda III or IV nodules from malignant nodules with a sensitivity of 91%, specificity of 68%, NPV of 96%, and PPV of 47% at 24% cancer prevalence (9). Meanwhile, the GSC method showed an overall false negative rate of 2% in a large new cohort study of Bethesda III or IV patients (67). GSC-benign nodules can be observed similarly to thyroid nodules with benign cytology (67). Therefore, individualized clinical factors and close long-term follow-up are recommended for the management of patients with ITNs (66). If the nodules are high risk with sonographic features, they should be given serious attention (64, 66). The PPV of oncocytic nodules was still low at 17% for Bethesda III nodules and 45% for Bethesda IV nodules. At the 1-year follow-up, only 22% of the thyroid nodules with benign GSC results grew during surveillance.

ThyroSeq serial next generation sequencing (NGS)-based molecular testing panels are potent and robust tools for diagnosing questionable thyroid nodules. Experts from the University of Pittsburgh Cancer Institute modified the ThyroSeq v3 panel into a DNA- and RNA-based NGS panel, incorporating a genomic classifier (GC) to distinguish malignant lesions from benign lesions (8). Complete ThyroSeq v3 is suitable for all common types of thyroid cancers and parathyroid lesions, with better efficacy than previous versions. A GC cutoff of 1.5 was identified to differentiate cancer from benign nodules with 93.9% sensitivity, 89.4% specificity, and 92.1% accuracy (68). In the FNA validation set, the sensitivity, specificity and accuracy of GC were 98.0%, 81.8%, and 90.9% (68). The analytical sensitivity, specificity, and robustness of ThyroSeq v3 GC have been successfully validated and clinically adopted in American, Southeast Asian, and Canadian cohorts (69, 71, 80, 81).

ThyroSeq v3 GC proposed a 3% false-negative rate (29) and helped reduce diagnostic surgery in up to 61% of patients with ITNs and in up to 82% of all benign ITNs. The performance of the ThyroSeq v3 GC in the Southeast Asian population was over 80% in all evaluated indices, which reduced to approximately 42% in diagnostic surgery (71). ThyroSeq positive-guided surgery for Bethesda IV nodules has increased cancer detection rates by four-fold, successfully identifying nearly all potentially aggressive malignancies. Over 80% of patients with negative ThyroSeq are able to safely undergo non-operative surveillance, remaining stable for 24.6 months (68).

Many studies have compared RNA-based Afirma GECs/GSCs with RNA-based ThyroSeq (70, 82). In principle, Afirma classifiers use ML to analyze gene expression data and build a binary diagnostic model that outputs results as either “suspicious” or “benign” (9). In contrast, ThyroSeq’s serial panels weigh mutant genes by number and category to calculate a risk grade using a fixed formula. This model defines results as “positive” or “negative” (8, 25). Afirma GSC and ThyroSeq v3 showed no significant differences in the benign call rate (53% vs. 61%), specificity (80% vs. 85%), and PPV (53% vs. 63%). Diagnostic thyroidectomy was avoided in 87 (51%) patients with benign GEC-benign nodules and 83 (49%) patients with ThyroSeq v3-negative nodules (70). Both Afirma GSC and ThyroSeq v3 are effective at ruling out malignancy in sonographically low-/intermediate-suspicion thyroid nodules but show limited diagnostic value for high-suspicion nodules (82).

## 4 Combination of US features and molecular testing for ITN diagnosis

Owing to increasing challenges in ITN diagnosis and management, combining US characteristics and molecular testing has been assumed to enhance diagnostic efficacy (31, 83). For example, incorporating the BRAF V600E mutation significantly enhanced diagnostic accuracy in detecting 511 ITNs across Korean, American, and Chinese TI-RADS systems. The AUC values were 0.773 vs. 0.735 (p<0.001) for K–TI-RADS, 0.809 vs. 0.778 (p<0.001) for ACR TI-RADS, and 0.815 vs. 0.783 (p<0.001) for C–TI-RADS at the cutoff for malignancy at grades 5, 5, and 4c, respectively (58). RAS-mutant ITNs often indicate a benign pathology (31), while RAS-mutant ITNs have higher rates of malignancy when multiple noncystic nodules or irregular borders are present. Excluding high-risk genetic markers for malignancy, the threshold for recommending surgical resection should be increased for ITNs (84). Some researchers hold negative opinions regarding molecular testing. Azaryan et al. retrospectively analyzed 237 Bethesda III/IV nodules by adding GSC results to the American Thyroid Association (ATA) risk stratification and ACR TI-RADS. They found no significant differences in ATA high-risk and TR5 nodules compared with ATA non-high-risk and TR1–4 nodules in terms of sensitivity, specificity, NPV, and PPV (85). In another multicenter study of ITNs using ThyroSeq v3 molecular testing results, neither the ATA nor TI-RADS US scoring systems further informed the risk of cancer/noninvasive follicular thyroid neoplasm with papillary-like nuclear features beyond that predicted by ThyroSeq v3 (81).

## 5 Challenges in ITN diagnosis

In radiomics, having a larger dataset and utilizing multiple imaging modalities improves the accuracy of AI-assisted diagnostic models. Specifically, AI-assisted models for diagnosis should be trained using ITN images, which would enhance their effectiveness. However, most models still have less performance stability and worse clinical applicability because of the limited amount of ITN data in retrospective studies or single-center datasets without external validation. The limited number of patients with ITN with confirmed pathology further constrains studies, as organizing multicenter collaboration remains challenging. Additionally, DL algorithms are popular for their automation and similarity to human cognitive processing, yet their “black box” nature makes it difficult for physicians to interpret their decisions. While AI-assisted models show better or comparable performance in ITN diagnosis compared with radiologists in most studies, they have shown inconsistent results in real-world settings (39). Given these complexities, AI should be seen as a supportive tool for clinicians rather than an independent diagnostic method.

Experts have increasingly recognized that molecular testing for ITNs can partially aid in preoperative diagnosis. An international study revealed an increasing trend in the use of molecular testing for diagnosing thyroid nodules from 2019 to 2022 (86), which accounted for only < 10%. The BRAF V600E mutation or the seven-gene panel for molecular testing is not commonly used in clinical practice in some countries. The GSC and ThyroSeq methods are primarily used and validated by institutions in the United States because they are expensive. Both the GSC and ThyroSeq testing panels lack support for multi-population data worldwide and long-term clinical visits. In other countries, multigene molecular testing panels remain in the exploration or preliminary stages, and other innovative molecular detection methods, such as QCIGISH, remain in the development stage. The use of multiple-gene testing panels from research to clinical practice is also influenced by local health agency policies, physician preferences, and patient privacy, despite the gradual acceptance of molecular testing before surgery and the decreased cost of molecular testing.

ITN diagnostic methods should utilize more recent technological advances. For example, super-resolution ultrasound imaging technology can help reveal the vascular structure, density, velocity, and direction of blood flow in tiny vessels while providing many new quantitative indices for medical analysis. ChatGPT, a text-based generative AI chatbot of large language models, nearly passed a radiology board-style examination without images despite having no radiology-specific pretraining (87). ChatGPT 4.0 demonstrated potential in enhancing diagnostic medical imaging, achieving an AUC of 0.83 and an accuracy of 84% (47). Furthermore, multicenter collaborations should be actively organized and conducted to solve sample size and applicability issues. Radiogenomics—the combination of radiomics and genomics—may also enhance diagnostic efficiency by elucidating the biological mechanisms underlying imaging results.

## 6 Conclusions

This review summarizes the latest publications on ITN diagnosis using AI-assisted ultrasound radiomics and genomic molecular testing over the last 5 years. Radiomics models have achieved comparable or superior performance than less experienced radiologists, enhancing diagnostic accuracy and reducing the number of FNA procedures. Genomic testing methods have proven to be effective in solving challenging ITN cases during preoperative diagnosis. Both radiomics and molecular testing, as supplementary tools, show positive effects in various research cohorts and require a large amount of data or long-term follow-up to support their clinical value. Continued exploration of these innovative diagnostic solutions is warranted.

## References

[1] Chmielik E Rusinek D Oczko-Wojciechowska M Jarzab M Krajewska J Czarniecka A. Heterogeneity of thyroid cancer. Pathobiology. (2018) 85:117–29. doi: 10.1159/000486422 29408820

[2] Siegel RL Giaquinto AN Jemal A. Cancer statistics, 2024. CA: A Cancer J Clin. (2024) 74:12–49. doi: 10.3322/caac.21820 38230766

[3] Tessler FN Middleton WD Grant EG Hoang JK Berland LL Teefey SA. Reporting and data system (TI-RADS): white paper of the ACR TI-RADS committee. J Am Coll Radiol. (2017) 14:587–95. doi: 10.1016/j.jacr.2017.01.046 28372962

[4] Cibas ES Ali SZ. The 2017 bethesda system for reporting thyroid cytopathology. Thyroid. (2017) 27:1341–6. doi: 10.1089/thy.2017.0500 29091573

[5] Ali SZ Baloch ZW Cochand-Priollet B Schmitt FC Vielh P VanderLaan PA. The 2023 bethesda system for reporting thyroid cytopathology. Thyroid®. (2023) 33(9):1039–44. doi: 10.1089/thy.2023.0141 37427847

[6] Haugen BR. 2015 American Thyroid Association Management Guidelines for Adult Patients with Thyroid Nodules and Differentiated Thyroid Cancer: What is new and what has changed? Cancer. (2017) 123:372–81. doi: 10.1002/cncr.30360 27741354

[7] Haugen BR Alexander EK Bible KC Doherty GM Mandel SJ Nikiforov YE. 2015 American thyroid association management guidelines for adult patients with thyroid nodules and differentiated thyroid cancer: the American thyroid association guidelines task force on thyroid nodules and differentiated thyroid cancer. Thyroid. (2016) 26:1–133. doi: 10.1089/thy.2015.0020 26462967 PMC4739132

[8] Nikiforova MN Mercurio S Wald AI Barbi de Moura M Callenberg K Santana-Santos L. Analytical performance of the ThyroSeq v3 genomic classifier for cancer diagnosis in thyroid nodules. Cancer. (2018) 124:1682–90. doi: 10.1002/cncr.31245 PMC5891361 29345728

[9] Patel KN Angell TE Babiarz J Barth NM Blevins T Duh Q-Y. Performance of a genomic sequencing classifier for the preoperative diagnosis of cytologically indeterminate thyroid nodules. JAMA Surg. (2018) 153(9):817–24. doi: 10.1001/jamasurg.2018.1153 PMC6583881 29799911

[10] Hoang JK Middleton WD Farjat AE Langer JE Reading CC Teefey SA. Reduction in thyroid nodule biopsies and improved accuracy with american college of radiology thyroid imaging reporting and data system. Radiology. (2018) 287:185–93. doi: 10.1148/radiol.2018172572 29498593

[11] Ha EJ Na DG Baek JH Sung JY Kim JH Kang SY. US fine-needle aspiration biopsy for thyroid Malignancy: diagnostic performance of seven society guidelines applied to 2000 thyroid nodules. Radiology. (2018) 287:893–900. doi: 10.1148/radiol.2018171074 29465333

[12] Wildman-Tobriner B Buda M Hoang JK Middleton WD Thayer D Short RG. Using artificial intelligence to revise ACR TI-RADS risk stratification of thyroid nodules: diagnostic accuracy and utility. Radiology. (2019) 292:112–9. doi: 10.1148/radiol.2019182128 31112088

[13] Grani G Lamartina L Ascoli V Bosco D Biffoni M Giacomelli L. Reducing the number of unnecessary thyroid biopsies while improving diagnostic accuracy: toward the “Right” TIRADS. J Clin Endocrinol Metab. (2019) 104:95–102. doi: 10.1210/jc.2018-01674 30299457

[14] Ruan JL Yang HY Liu RB Liang M Han P Xu XL. Fine needle aspiration biopsy indications for thyroid nodules: compare a point-based risk stratification system with a pattern-based risk stratification system. Eur Radiol. (2019) 29:4871–8. doi: 10.1007/s00330-018-5992-z 30715590

[15] Gillies RJ Kinahan PE Hricak H. Radiomics: images are more than pictures, they are data. Radiology. (2016) 278:563–77. doi: 10.1148/radiol.2015151169 PMC4734157 26579733

[16] Wu G-G Lv W-Z Yin R Xu J-W Yan Y-J Chen R-X. Deep learning based on ACR TI-RADS can improve the differential diagnosis of thyroid nodules. Front Oncol. (2021) 11:575166. doi: 10.3389/fonc.2021.575166 33987082 PMC8111071

[17] Liang X Huang Y Cai Y Liao J Chen Z Computer-Aided-Diagnosis-System A. and thyroid imaging reporting and data system for dual validation of ultrasound-guided fine-needle aspiration of indeterminate thyroid nodules. Front Oncol. (2021) 11:611436. doi: 10.3389/fonc.2021.611436 34692466 PMC8529148

[18] Gong Zj Xin J Yin J Wang B Li X Yang Hx. Diagnostic value of artificial intelligence-assistant diagnostic system combined with contrast-enhanced ultrasound in thyroid TI-RADS 4 nodules. J Ultrasound Med. (2023) 42:1527–35. doi: 10.1002/jum.16170 36723397

[19] Ren J-Y Lv W-Z Wang L Zhang W Ma Y-Y Huang Y-Z. Dual-modal radiomics nomogram based on contrast-enhanced ultrasound to improve differential diagnostic accuracy and reduce unnecessary biopsy rate in ACR TI-RADS 4–5 thyroid nodules. Cancer Imaging. (2024) 24(1):17. doi: 10.1186/s40644-024-00661-3 38263209 PMC10807093

[20] Moraes PHM Takahashi MS Vanderlei FAB Schelini MV Chacon DA Tavares MR. Multiparametric ultrasound evaluation of the thyroid: elastography as a key tool in the risk prediction of undetermined nodules (Bethesda III and IV)—Histopathological correlation. Ultrasound Med Biol. (2021) 47:1219–26. doi: 10.1016/j.ultrasmedbio.2021.01.019 33583638

[21] D’Andréa G Gal J Mandine L Dassonville O Vandersteen C Guevara N. Application of machine learning methods to guide patient management by predicting the risk of Malignancy of Bethesda III-V thyroid nodules. Eur J Endocrinol. (2023) 188(3):lvad017. doi: 10.1093/ejendo/lvad017 36799885

[22] Li Y Liu Y Xiao J Yan L Yang Z Li X. Clinical value of artificial intelligence in thyroid ultrasound: a prospective study from the real world. Eur Radiol. (2023) 33:4513–23. doi: 10.1007/s00330-022-09378-y 36622410

[23] Park VY Lee E Lee HS Kim HJ Yoon J Son J. Combining radiomics with ultrasound-based risk stratification systems for thyroid nodules: an approach for improving performance. Eur Radiol. (2020) 31:2405–13. doi: 10.1007/s00330-020-07365-9 33034748

[24] Zhao CK Ren TT Yin YF Shi H Wang HX Zhou BY. A comparative analysis of two machine learning-based diagnostic patterns with thyroid imaging reporting and data system for thyroid nodules: diagnostic performance and unnecessary biopsy rate. Thyroid. (2021) 31:470–81. doi: 10.1089/thy.2020.0305 32781915

[25] Nikiforov YE Carty SE Chiosea SI Coyne C Duvvuri U Ferris RL. Impact of the multi-gene thyroSeq next-generation sequencing assay on cancer diagnosis in thyroid nodules with atypia of undetermined significance/follicular lesion of undetermined significance cytology. Thyroid. (2015) 25:1217–23. doi: 10.1089/thy.2015.0305 PMC4652198 26356635

[26] Eszlinger M Piana S Moll A Bösenberg E Bisagni A Ciarrocchi A. Molecular testing of thyroid fine-needle aspirations improves presurgical diagnosis and supports the histologic identification of minimally invasive follicular thyroid carcinomas. Thyroid. (2015) 25:401–9. doi: 10.1089/thy.2014.0362 25629769

[27] Scheffel RS Dora JM Maia AL. BRAF mutations in thyroid cancer. Curr Opin Oncol. (2022) 34:9–18. doi: 10.1097/cco.0000000000000797 34636352

[28] An JH Song KH Kim SK Park KS Yoo YB Yang JH. RAS mutations in indeterminate thyroid nodules are predictive of the follicular variant of papillary thyroid carcinoma. Clin Endocrinol (Oxf). (2015) 82:760–6. doi: 10.1111/cen.12579 25109485

[29] Steward DL Carty SE Sippel RS Yang SP Sosa JA Sipos JA. Performance of a multigene genomic classifier in thyroid nodules with indeterminate cytology. JAMA Oncol. (2019) 5(2):204–12. doi: 10.1001/jamaoncol.2018.4616 PMC6439562 30419129

[30] Stewardson P Eszlinger M Wu J Khalil M Box A Perizzolo M. Prospective validation of thyroSPEC molecular testing of indeterminate thyroid nodule cytology following diagnostic pathway optimization. Thyroid®. (2023) 33:1423–33. doi: 10.1089/thy.2023.0255 37742115

[31] Torrecillas V Sharma A Neuberger K Abraham D. Utility of mutational analysis for risk stratification of indeterminate thyroid nodules in a real-world setting. Clin Endocrinol. (2021) 96:637–45. doi: 10.1111/cen.14601 34605038

[32] Talmor G Badash I Zhou S Kim YJ Kokot NC Hsueh W. Association of patient characteristics, ultrasound features, and molecular testing with Malignancy risk in Bethesda III–V thyroid nodules. Laryngoscope Invest Otolaryngol. (2022) 7:1243–50. doi: 10.1002/lio2.847 PMC9392397 36000058

[33] Alyusuf EY Alhmayin L Albasri E Enani J Altuwaijri H Alsomali N. Ultrasonographic predictors of thyroid cancer in Bethesda III and IV thyroid nodules. Front Endocrinol. (2024) 15:1326134. doi: 10.3389/fendo.2024.1326134 PMC10884110 38405143

[34] Shin JH Baek JH Chung J Ha EJ Kim JH Lee YH. Ultrasonography diagnosis and imaging-based management of thyroid nodules: revised Korean society of thyroid radiology consensus statement and recommendations. Korean J Radiol. (2016) 17:370–95. doi: 10.3348/kjr.2016.17.3.370 PMC4842857 27134526

[35] Zhou J Yin L Wei X Zhang S Song Y Luo B. 2020 Chinese guidelines for ultrasound Malignancy risk stratification of thyroid nodules: the C-TIRADS. Endocrine. (2020) 70:256–79. doi: 10.1007/s12020-020-02441-y 32827126

[36] Huang X Wu Z Zhou A Min X Qi Q Zhang C. Nomogram combining radiomics with the American college of radiology thyroid imaging reporting and data system can improve predictive performance for Malignant thyroid nodules. Front Oncol. (2021) 11:737847. doi: 10.3389/fonc.2021.737847 34722287 PMC8550451

[37] Peng S Liu Y Lv W Liu L Zhou Q Yang H. Deep learning-based artificial intelligence model to assist thyroid nodule diagnosis and management: a multicentre diagnostic study. Lancet Digit Health. (2021) 3:e250–9. doi: 10.1016/s2589-7500(21)00041-8 33766289

[38] Gild ML Chan M Gajera J Lurie B Gandomkar Z Clifton-Bligh RJ. Risk stratification of indeterminate thyroid nodules using ultrasound and machine learning algorithms. Clin Endocrinol. (2021) 96:646–52. doi: 10.1111/cen.14612 34642976

[39] Han M Ha EJ Park JH. Computer-aided diagnostic system for thyroid nodules on ultrasonography: diagnostic performance based on the thyroid imaging reporting and data system classification and dichotomous outcomes. Am J Neuroradiol. (2021) 42:559–65. doi: 10.3174/ajnr.A6922 PMC7959443 33361374

[40] Luo P Fang Z Zhang P Yang Y Zhang H Su L. Radiomics score combined with ACR TI-RADS in discriminating benign and Malignant thyroid nodules based on ultrasound images: A retrospective study. Diagnostics. (2021) 11(6):1011. doi: 10.3390/diagnostics11061011 34205943 PMC8229428

[41] Tang J Jiang S Ma J Xi X Li H Wang L. Nomogram based on radiomics analysis of ultrasound images can improve preoperative BRAF mutation diagnosis for papillary thyroid microcarcinoma. Front Endocrinol. (2022) 13:915135. doi: 10.3389/fendo.2022.915135 PMC9437521 36060960

[42] Chen L Chen M Li Q Kumar V Duan Y Wu KA. Machine learning-assisted diagnostic system for indeterminate thyroid nodules. Ultrasound Med Biol. (2022) 48:1547–54. doi: 10.1016/j.ultrasmedbio.2022.03.020 35660106

[43] Kim Y-J Choi Y Hur S-J Park K-S Kim H-J Seo M. Deep convolutional neural network for classification of thyroid nodules on ultrasound: Comparison of the diagnostic performance with that of radiologists. Eur J Radiol. (2022) 152:110335. doi: 10.1016/j.ejrad.2022.110335 35512512

[44] Keutgen XM Li H Memeh K Conn Busch J Williams J Lan L. A machine-learning algorithm for distinguishing Malignant from benign indeterminate thyroid nodules using ultrasound radiomic features. J Med Imaging. (2022) 9(3):034501. doi: 10.1117/1.Jmi.9.3.034501 PMC9133922 35692282

[45] Wang B Wan Z Li C Zhang M Shi Y Miao X. Identification of benign and Malignant thyroid nodules based on dynamic AI ultrasound intelligent auxiliary diagnosis system. Front Endocrinol. (2022) 13:1018321. doi: 10.3389/fendo.2022.1018321 PMC9551607 36237194

[46] Zhou L Zheng L-l Zhang C-j Wei H-f Xu L-l Zhang M-r. Comparison of S-Detect and thyroid imaging reporting and data system classifications in the diagnosis of cytologically indeterminate thyroid nodules. Front Endocrinol. (2023) 14:1098031. doi: 10.3389/fendo.2023.1098031 PMC9902707 36761203

[47] Wu S-H Tong W-J Li M-D Hu H-T Lu X-Z Huang Z-R. Collaborative enhancement of consistency and accuracy in US diagnosis of thyroid nodules using large language models. Radiology. (2024) 310(3):e232255. doi: 10.1148/radiol.232255 38470237

[48] Guo SY Zhou P Zhang Y Jiang LQ Zhao YF. Exploring the value of radiomics features based on B-mode and contrast-enhanced ultrasound in discriminating the nature of thyroid nodules. Front Oncol. (2021) 11:738909. doi: 10.3389/fonc.2021.738909 34722288 PMC8551634

[49] Zhou T Xu L Shi J Zhang Y Lin X Wang Y. US of thyroid nodules: can AI-assisted diagnostic system compete with fine needle aspiration? Eur Radiol. (2023) 34:1324–33. doi: 10.1007/s00330-023-10132-1 37615763

[50] Tong W-J Wu S-H Cheng M-Q Huang H Liang J-Y Li C-Q. Integration of artificial intelligence decision aids to reduce workload and enhance efficiency in thyroid nodule management. JAMA Netw Open. (2023) 6(5):e2313674. doi: 10.1001/jamanetworkopen.2023.13674 37191957 PMC10189570

[51] Radzina M Ratniece M Putrins DS Saule L Cantisani V. Performance of contrast-enhanced ultrasound in thyroid nodules: review of current state and future perspectives. Cancers (Basel). (2021) 13(21):5469. doi: 10.3390/cancers13215469 34771632 PMC8582579

[52] Sidhu PS Cantisani V Dietrich CF Gilja OH Saftoiu A Bartels E. The EFSUMB guidelines and recommendations for the clinical practice of contrast-enhanced ultrasound (CEUS) in non-hepatic applications: update 2017 (Long version). Ultraschall Med. (2018) 39:e2–e44. doi: 10.1055/a-0586-1107 29510439

[53] Yongfeng Z Ping Z Hong P Wengang L Yan Z. Superb microvascular imaging compared with contrast-enhanced ultrasound to assess microvessels in thyroid nodules. J Med Ultrason (2001). (2020) 47:287–97. doi: 10.1007/s10396-020-01011-z 32125575

[54] Moraes PHM Takahashi MS Vanderlei FAB Schelini MV Chacon DA Tavares MR. Multiparametric ultrasound evaluation of the thyroid: elastography as a key tool in the risk prediction of undetermined nodules (Bethesda III and IV)-histopathological correlation. Ultrasound Med Biol. (2021) 47:1219–26. doi: 10.1016/j.ultrasmedbio.2021.01.019 33583638

[55] Hang J Li F Qiao XH Ye XH Li A Du LF. Combination of maximum shear wave elasticity modulus and TIRADS improves the diagnostic specificity in characterizing thyroid nodules: A retrospective study. Int J Endocrinol. (2018) 2018:4923050. doi: 10.1155/2018/4923050 30402095 PMC6198550

[56] Nikiforov YE Carty SE Chiosea SI Coyne C Duvvuri U Ferris RL. Highly accurate diagnosis of cancer in thyroid nodules with follicular neoplasm/suspicious for a follicular neoplasm cytology by ThyroSeq v2 next-generation sequencing assay. Cancer. (2014) 120:3627–34. doi: 10.1002/cncr.29038 PMC7737376 25209362

[57] Goldner WS Angell TE McAdoo SL Babiarz J Sadow PM Nabhan FA. Molecular variants and their risks for Malignancy in cytologically indeterminate thyroid nodules. Thyroid. (2019) 29:1594–605. doi: 10.1089/thy.2019.0278 PMC6864764 31469053

[58] Lin Y Cheng Y Zhang Y Ren X Li J Shi H. The value of Korean, American, and Chinese ultrasound risk stratification systems combined with BRAF(V600E) mutation for detecting papillary thyroid carcinoma in cytologically indeterminate thyroid nodules. Endocrine. (2023) 84(2):549–59. doi: 10.1007/s12020-023-03586-2 37940765

[59] Zhou Y Wu X Zhang Y Li Z Ge X Chen H. Performance of multigene testing in cytologically indeterminate thyroid nodules and molecular risk stratification. PeerJ. (2023) 11:e16054. doi: 10.7717/peerj.16054 37744220 PMC10512961

[60] Daniels KE Xu J Liu J-B Chen X Huang K Patel J. Diagnostic value of TI-RADS classification system and next generation genetic sequencing in indeterminate thyroid nodules. Acad Radiol. (2021) 28:1685–91. doi: 10.1016/j.acra.2020.07.037 32839097

[61] Tolaba N Spedalletti Y Bazzoni P Galindez M Cerioni V Santillan C. Testing of mutations on thyroid nodules with indeterminate cytology: A prospective study of 112 patients in Argentina. Endocrinol Diabetes y Nutrición (English ed.). (2022) 69:122–30. doi: 10.1016/j.endien.2022.02.002 35256055

[62] Hu C Jing W Chang Q Zhang Z Liu Z Cao J. Risk stratification of indeterminate thyroid nodules by novel multigene testing: a study of Asians with a high risk of Malignancy. Mol Oncol. (2022) 16:1680–93. doi: 10.1002/1878-0261.13205 PMC9019878 35247035

[63] Alexander EK Kennedy GC Baloch ZW Cibas ES Chudova D Diggans J. Preoperative diagnosis of benign thyroid nodules with indeterminate cytology. N Engl J Med. (2012) 367:705–15. doi: 10.1056/NEJMoa1203208 22731672

[64] Endo M Porter K Long C Azaryan I Phay JE Ringel MD. Features of cytologically indeterminate molecularly benign nodules treated with surgery. J Clin Endocrinol Metab. (2020) 105:e3971–80. doi: 10.1210/clinem/dgaa506 PMC7497819 32772084

[65] Hu MI Waguespack SG Dosiou C Ladenson PW Livhits MJ Wirth LJ. Afirma genomic sequencing classifier and xpression atlas molecular findings in consecutive bethesda III-VI thyroid nodules. J Clin Endocrinol Metab. (2021) 106:2198–207. doi: 10.1210/clinem/dgab304 PMC8277199 34009369

[66] White MK Thedinger WB Dhingra JK. Long-term follow-up of cytologically indeterminate thyroid nodules found benign on molecular testing: A validation study. OTO Open. (2022) 6(1):2473974X221083542. doi: 10.1177/2473974x221083542 PMC8935552 35321424

[67] Ahmadi S Kotwal A Bikas A Xiang P Goldner W Patel A. Outcomes of cytologically indeterminate thyroid nodules managed with genomic sequencing classifier. J Clin Endocrinol Metab. (2024) 109(12):e2231–e2239. doi: 10.1210/clinem/dgae112 38415829

[68] Carty SE Ohori NP Hilko DA McCoy KL French EK Manroa P. The clinical utility of molecular testing in the management of thyroid follicular neoplasms (Bethesda IV nodules). Ann Surg. (2020) 272:621–7. doi: 10.1097/sla.0000000000004130 32773640

[69] Desai D Lepe M Baloch ZW Mandel SJ. ThyroSeq v3 for Bethesda III and IV: An institutional experience. Cancer Cytopathol. (2020) 129:164–70. doi: 10.1002/cncy.22362 33030808

[70] Livhits MJ Zhu CY Kuo EJ Nguyen DT Kim J Tseng C-H. Effectiveness of molecular testing techniques for diagnosis of indeterminate thyroid nodules. JAMA Oncol. (2021) 7(1):70–7. doi: 10.1001/jamaoncol.2020.5935 PMC7729582 33300952

[71] Yang SP Nga ME Bundele MM Chiosea SI Tan SH Lum JHY. Performance of a multigene genomic classifier and clinical parameters in predicting Malignancy in a Southeast Asian cohort of patients with cytologically indeterminate thyroid nodules. Cancer Cytopathol. (2024) 132(5):309–19. doi: 10.1002/cncy.22796 38319805

[72] Xu H Zhang Y Wu H Zhou N Li X Pineda JP. High diagnostic accuracy of epigenetic imprinting biomarkers in thyroid nodules. J Clin Oncol. (2023) 41:1296–306. doi: 10.1200/jco.22.00232 PMC9937101 36378996

[73] Glass RE Levy JJ Motanagh SA Vaickus LJ Liu X. Atypia of undetermined significance in thyroid cytology: Nuclear atypia and architectural atypia are associated with different molecular alterations and risks of Malignancy. Cancer Cytopathol. (2021) 129:966–72. doi: 10.1002/cncy.22495 34399035

[74] Rossi M Buratto M Tagliati F Rossi R Lupo S Trasforini G. Relevance of BRAF(V600E) mutation testing versus RAS point mutations and RET/PTC rearrangements evaluation in the diagnosis of thyroid cancer. Thyroid. (2015) 25:221–8. doi: 10.1089/thy.2014.0338 PMC4322031 25333496

[75] Sfreddo HJ Koh ES Zhao K Swartzwelder CE Untch BR Marti JL. RAS-mutated cytologically indeterminate thyroid nodules: prevalence of Malignancy and behavior under active surveillance. Thyroid®. (2024) 34(4):450–9. doi: 10.1089/thy.2023.0544 PMC11971614 38407967

[76] Baltzer PAT Wu H Zhang B Cai G Li J Gu X. American College of Radiology thyroid imaging report and data system combined with K-RAS mutation improves the management of cytologically indeterminate thyroid nodules. PloS One. (2019) 14(7):e0219383. doi: 10.1371/journal.pone.0219383 31295281 PMC6622496

[77] Jurkiewicz M Cimic A Murty VV Kuo JH Hsiao S Fazlollahi L. Detection of STRN-ALK fusion in thyroid nodules with indeterminate cytopathology facilitates papillary thyroid cancer diagnosis. Diagn Cytopathol. (2020) 49(4):E146–E151. doi: 10.1002/dc.24647 33085842

[78] Morariu EM McCoy KL Chiosea SI Nikitski AV Manroa P Nikiforova MN. Clinicopathologic characteristics of thyroid nodules positive for theTHADA-IGF2BP3Fusion on preoperative molecular analysis. Thyroid. (2021) 31:1212–8. doi: 10.1089/thy.2020.0589 33487086

[79] Shen R Cheng T Xu C Yung RC Bao J Li X. Novel visualized quantitative epigenetic imprinted gene biomarkers diagnose the Malignancy of ten cancer types. Clin Epigenet. (2020) 12(1):71. doi: 10.1186/s13148-020-00861-1 PMC7245932 32448196

[80] Chen T Gilfix BM Rivera J Sadeghi N Richardson K Hier MP. The role of the thyroSeq v3 molecular test in the surgical management of thyroid nodules in the Canadian public health care setting. Thyroid. (2020) 30:1280–7. doi: 10.1089/thy.2019.0539 32242511

[81] Figge JJ Gooding WE Steward DL Yip L Sippel RS Yang SP. Do ultrasound patterns and clinical parameters inform the probability of thyroid cancer predicted by molecular testing in nodules with indeterminate cytology? Thyroid. (2021) 31:1673–82. doi: 10.1089/thy.2021.0119 PMC8917891 34340592

[82] Hu TX Nguyen DT Patel M Beckett K Douek M Masamed R. The effect modification of ultrasound risk classification on molecular testing in predicting the risk of Malignancy in cytologically indeterminate thyroid nodules. Thyroid. (2022) 32:905–16. doi: 10.1089/thy.2021.0659 35611970

[83] Zahid A Shafiq W Nasir KS Loya A Abbas Raza S Sohail S. Malignancy rates in thyroid nodules classified as Bethesda categories III and IV; a subcontinent perspective. J Clin Transl Endocrinol. (2021) 23:100250. doi: 10.1016/j.jcte.2021.100250 33643850 PMC7887641

[84] Bernuy MCCR Tessnow A Hussain I. Indeterminate thyroid nodules with RAS mutations have higher rates of Malignancy when multiple non-cystic nodules or irregular borders are present. J Endocr Soc. (2021) 5:A862–3. doi: 10.1210/jendso/bvab048.1761

[85] Azaryan I Endo M Sipos JA Ma J Peng J Nabhan F. Ultrasound features and performance of afirma gene sequencing classifier in cytologically indeterminate thyroid nodules. J Endocr Soc. (2024) 8(3):bvae010. doi: 10.1210/jendso/bvae010 38348302 PMC10859306

[86] Medas F Dobrinja C Al-Suhaimi EA Altmeier J Anajar S Arikan AE. Effect of the COVID-19 pandemic on surgery for indeterminate thyroid nodules (THYCOVID): a retrospective, international, multicentre, cross-sectional study. Lancet Diabetes Endocrinol. (2023) 11:402–13. doi: 10.1016/s2213-8587(23)00094-3 PMC10147315 37127041

[87] Bhayana R Krishna S Bleakney RR. Performance of chatGPT on a radiology board-style examination: insights into current strengths and limitations. Radiology. (2023) 307(5):e230582. doi: 10.1148/radiol.230582 37191485

